# Urine biomarkers give early prediction of acute kidney injury and outcome after out-of-hospital cardiac arrest

**DOI:** 10.1186/s13054-016-1503-2

**Published:** 2016-10-05

**Authors:** Sigrid Beitland, Bård Endre Waldum-Grevbo, Espen Rostrup Nakstad, Jens-Petter Berg, Anne-Marie Siebke Trøseid, Berit Sletbakk Brusletto, Cathrine Brunborg, Geir Øystein Andersen, Kjetil Sunde

**Affiliations:** 1 Institute of Clinical Medicine, University of Oslo, Oslo, Norway; 2 Department of Anaesthesiology, Oslo University Hospital, Oslo, Norway; 3 Department of Nephrology, Oslo University Hospital, Oslo, Norway; 4 Norwegian National Unit for CBRNE Medicine, Oslo University Hospital, Oslo, Norway; 5 Department of Medical Biochemistry, Oslo University Hospital, Oslo, Norway; 6 Oslo Centre for Biostatistics and Epidemiology, Oslo University Hospital, Oslo, Norway; 7 Department of Cardiology, Oslo University Hospital, Oslo, Norway

**Keywords:** Acute kidney injury, Cardiac arrest, Biomarker, Outcome, Prognosis

## Abstract

**Background:**

Post-resuscitation care after out-of-hospital cardiac arrest (OHCA) is challenging due to the threat of organ failure and difficult prognostication. Our aim was to examine whether urine biomarkers could give an early prediction of acute kidney injury (AKI) and outcome.

**Methods:**

This was a prospective observational study of comatose OHCA patients at Oslo University Hospital Ullevål, Norway. Risk factors were clinical parameters and biomarkers measured in spot urine (cystatin C, neutrophil gelatinase-associated lipocalin (NGAL) and the product of tissue inhibitor of metalloproteinase 2 (TIMP-2) and insulin-like growth factor-binding protein 7 (IGFBP7)) at admission and day 3. Outcome variables were AKI within 3 days using the Kidney Disease Improving Global Outcomes definition, 6-month mortality, and poor neurological outcome (PNO) defined as cerebral performance category 3–5.

**Results:**

Among 195 included patients (85 % males, mean age 60 years), 88 (45 %) died, 96 (49 %) had PNO, and 88 (45 %) developed AKI. In univariate analysis, increased urine cystatin C and NGAL concentration sampled at admission and day 3 were independent risk factors for AKI, mortality and PNO. Increased urine TIMP-2 × IGFBP7 levels was associated with AKI only at admission. In multivariate analyses combining clinical parameters and biomarker concentrations, the area under the receiver operating characteristics curve (AuROC) with 95 % confidence interval (CI) were 0.774 (0.700–0.848), 0.812 (0.751–0.873), and 0.819 (0.759–0.878) for AKI, mortality and PNO, respectively.

**Conclusions:**

In comatose OHCA patients, urine levels of cystatin C and NGAL at admission and day 3 were independent risk factors for AKI, 6-month mortality and PNO.

**Trial registration:**

Clinicaltrials.gov NCT01239420. Registered 10 November 2010.

**Electronic supplementary material:**

The online version of this article (doi:10.1186/s13054-016-1503-2) contains supplementary material, which is available to authorized users.

## Background

Out-of-hospital cardiac arrest (OHCA) is a major health problem, with an incidence rate in Europe of 84 per 100,000 inhabitants per year [1]. Among those admitted to hospital with return of spontaneous circulation (ROSC), mortality at 30 days, or to hospital discharge is on average 58 % [1], but with large variations across sites. Due to the reperfusion injury seen in the post cardiac arrest (CA) syndrome [2, 3], these patients are disposed to develop multiple organ failure [4], with acute kidney injury (AKI) affecting about half of the survivors [5, 6]. Even though most organ functions recover, some patients suffer long-time disability with poor neurological outcome (PNO) [7]. A huge challenge for clinicians is the lack of reliable predictors of AKI, mortality, and neurological outcome after OHCA. An early diagnostic and/or prognostic biomarker could potentially optimize targeted post-resuscitation care and reduce the burden of futile treatment to patients, relatives and the healthcare system [8].

There are many candidate biomarkers that aim to predict AKI and prognosis after CA, but none of these have discriminating power high enough to be recommended for routine use [9, 10]. Promising biomarkers of AKI are cystatin C [11], neutrophil gelatinase-associated lipocalin (NGAL) [12], tissue inhibitor of metalloproteinase 2 (TIMP-2), and insulin-like growth factor-binding protein 7 (IGFBP7) [13]. Recent studies of CA patients revealed that NGAL measured in blood was a predictor of AKI [14], mortality [14, 15], and neurological outcome [14]. In one of the studies, enrolment serum NGAL actually performed better than neuron-specific enolase and S100B in predicting survival to hospital discharge [14]. However, we lack data on the diagnostic and prognostic utility of the AKI biomarkers cystatin C, NGAL, TIMP-2, and IGFBP7 measured in urine early after OHCA.

The primary aim of this study was to examine the ability of urine biomarkers to predict AKI, mortality and PNO after OHCA. The secondary aim was to find the optimal biomarker and sampling time, and to investigate if the discriminating power was improved in models combining clinical parameters and biomarker concentrations.

## Methods

### Study design and setting

Patients were consecutively enrolled in this prospective study of OHCA patients as an a priori planned substudy of the yet not published Norwegian Cardiorespiratory Arrest Study (NORCAST) (NCT01239420). The primary aim of the NORCAST study was to assess early predictors of patient outcome after OHCA. Oslo University Hospital Ullevål is a community hospital for approximately 200,000 people, and a regional hospital for 1.4 million people in Norway, with around 45,000 admissions per year. The Regional Committee for Medical Research Ethics of Eastern and Southern Norway approved the study.

### Study population

Adult (≥18 years) comatose (Glasgow Coma Scale ≤8 at admission) OHCA patients with ROSC admitted between 8 September 2010 and 13 January 2014 were included. Patients with known chronic kidney disease (CKD), or who died within 24 h of intensive care unit (ICU) stay, or for some reason did not receive active treatment, were excluded. Patients were treated according to our own standard operating procedure (SOP) for OHCA, including the use of targeted temperature management (TTM), with target set at 33 °C for 24 h. Patients were followed in detail during their hospital stay until an extensive 6-month post-arrest consultation.

### Study definitions

OHCA was defined as the absence of spontaneous respiration in a comatose patient receiving cardiopulmonary resuscitation (CPR). ROSC was identified as sustained electrical activity on the electrocardiogram generating a palpable pulse. AKI and CKD were classified according to Kidney Disease Improving Global Outcomes (KDIGO) guidelines [16, 17], but only data from the first 3 days of ICU stay were assessed. In the definition of AKI the patients’ steady-state creatinine concentrations prior to CA remain unknown, and we used relative changes from creatinine levels at admission. The worst of the serum creatinine and urine output criteria was considered, and all patients undergoing renal replacement therapy (RRT) were classified as stage 3. PNO was defined as a cerebral performance category 3–5 [7]. Severity of illness was assessed using the Simplified Acute Physiology Score (SAPS) II [18], and the extent of organ failure was considered utilizing the Sequential Organ Failure Assessment (SOFA) score [19].

### Data collection

Baseline characteristics such as age, weight, sex, and prior medical history were prospectively collected. Traditional prehospital data following the Utstein criteria [20] were obtained from the paramedic records and CA registry. Patient data from the first days was collected from the ICU charts, including blood sample results from routine laboratory investigations, fluid balance (perspiration not included), and severity of illness scores (SOFA and SAPS II). Additional data on mortality and neurological outcome were obtained during an extensive consultation 6 months post-arrest.

### Biochemical sampling and analyses

Spot urine samples were collected from urine catheters at admission (0 to 6 h post-arrest) and on day 3 after the OHCA. Samples were stored in a refrigerator for up to 72 h before being frozen at –70 °C. After thawing, samples were centrifuged for 5 min at 20 °C and 500 RCF, aliquoted, and refrozen. Thereafter, the urine samples were re-thawed and identically re-centrifuged before they were diluted 1:200 and run in duplicate according to the manufacturers’ instructions. Cystatin C and NGAL were quantified using Bio-Plex Pro RBM Human Kidney Toxicity Assays panel 2 on the Bio-Plex 200 system (Bio-Rad Laboratories, Hercules, CA, USA). The concentrations of TIMP-2 and IGFBP7 were measured using the NephroCheck™ Test (Astute Medical, San Diego, CA, USA), calculating the product of both biomarker concentrations (TIMP-2 × IGFBP7). For all biomarkers, results below the lower range were set as 0, and results above the upper range were set as 100,000. A pilot study revealed that the studied biomarkers in urine were stable when they were stored in a refrigerator for up to 72 h prior to freezing, and when centrifuged after thawing [21].

### Statistical methods

Data are presented as number (percentage), median (interquartile range (IQR)) or mean (standard deviation (SD)). Univariate analyses were performed using the Pearson’s Chi square test and Fisher’s exact test when appropriate. The association between potential risk factors and the outcomes AKI, mortality and PNO were quantified by odds ratio (OR) with 95 % confidence interval (CI). Variables with *p* < 0.25 in the univariate analyses were considered candidates for the multivariate model if they had less than 15 % missing data. Independent risk factors were identified using a multivariate logistic regression model and a manual backward stepwise elimination procedure. Multivariate analyses were preceded by estimation of correlation between risk factors. The predictive accuracy of the models was assessed by calibration and discrimination. Calibration was evaluated by the Hosmer and Lemeshow goodness-of-fit test. A statistically non-significant Hosmer and Lemeshow result (*p* > 0.05) suggests that the model predicts accurately on average. Discrimination was evaluated by analysis of the area under the receiver operating characteristics curve (ROC) curve, and acceptable discriminatory capability was defined as an area under the ROC curve (AuROC) above 0.7. Chi-square tests for equality of AuROCs were performed using Stata 14 (StataCorp, College Station, TX, USA); all other statistical analyses were performed using SPSS 21 for Windows (IBM SPSS, Chicago, IL, USA). Two-sided *p* values less than 0.05 were considered statistically significant. Patients without recorded body weight were assumed to be 70 kg if female and 80 kg if male in the calculation of hourly urine output. There were some additional missing data that were handled using only available data.

## Results

### Patient characteristics and event rates

**Fig. 1:**
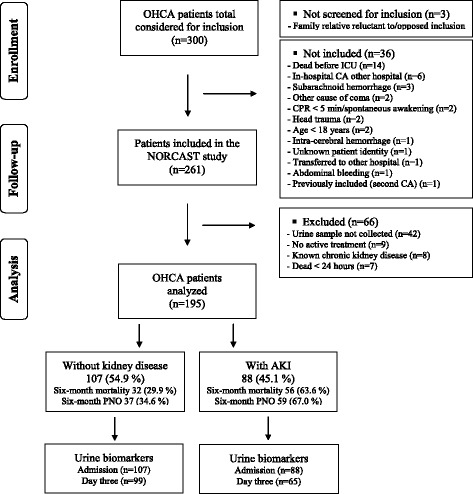
Flow chart of the study. *AKI* acute kidney injury, *OHCA* out-of-hospital cardiac arrest, *CA* cardiac arrest, *CPR* cardiopulmonary resuscitation, *ICU* intensive care unit, *NORCAST* Norwegian Cardiorespiratory Arrest Study, *PNO* poor neurological outcome (defined as cerebral performance category 3–5)

Of 300 OHCA patients eligible during the study period, 261were included in the NORCAST study. Altogether 66 patients were excluded from this substudy due to different reasons (Fig. 1).

In the total cohort of 195 included patients, 165 (85 %) were male and the mean age was 60 (±14) years. Overall 6-month outcome revealed that 88 (45 %) died and 96 (49 %) had PNO (Table 1). In total, 88 patients (45 %) developed AKI;﻿ 52 (27 %), 23 (12 %), and 13 (7 %) with stage 1, 2, and 3, respectively. Urine samples were collected from all 195 patients at admission and 164 (84 %) patients at day 3.

**Table 1:** Univariate analysis of risk factors for acute kidney injury in resuscitated, comatose out-of- hospital cardiac arrest patients

	Without AKI (*n* = 107)	With AKI (*n* = 88)	Risk factor for AKI	Crude OR (95 % CI) for AKI	*p* value
Baseline data					
Age, years	60.0 ± 13.7	60.2 ± 13.4	Age ≥60 years	0.90 (0.51–1.58)	0.710
Weight, kg^a^ (*n* = 166)	80.0 (75.0–90.0)	85.0 (80.0–94.5)	Weight ≥85 kg	1.73 (0.93–3.20)	0.083
Male sex	92 (86.0)	73 (83.0)	Female sex	1.26 (0.58–2.75)	0.560
Witnessed CA^a^ (*n* = 194)	98 (92.5)	71 (80.7)	Unwitnessed CA	2.93 (1.20–7.17)	0.015
Bystander CPR	96 (89.7)	75 (85.2)	Not bystander CPR	1.51 (0.64–3.57)	0.342
ROSC time, min^a^ (*n* = 158)	22.0 (15.0–29.0)	30.0 (20.0–42.5)	Time to ROSC ≥25 min	2.16 (1.13–4.11)	0.018
Initial VF/VT^a^ (*n* = 193)	76 (71.0)	52 (59.1)	Not initial VF/VT	1.71 (0.93–3.11)	0.081
SAPS II, score	68.2 ± 10.1	73.1 ± 10.3	SAPS II score ≥69	1.92 (1.08–3.42)	0.026
Admission day					
Diuresis, L/day	2.26 (1.82–3.28)	1.81 (1.43–2.45)	Diuresis <1.93 L/day	3.69 (2.04–6.70)	<0.001
Fluid balance, L/day	4.01 (2.79–5.77)	4.74 (3.50–6.30)	Fluid balance ≥4.45 L/day	1.49 (0.84–2.62)	0.169
S-Creatinine, μmol/L	94.0 (81.3–105.0)	107.0 (94.0–140.0)	S-Creatinine ≥101 μmol/L	5.18 (2.80–9.59)	<0.001
S-Urea; mmol/L	6.3 (5.2–7.5)	7.3 (5.8–9.6)	S-Urea ≥6.7 mmol/L	2.74 (1.53–4.91)	0.001
B-HCO_3_^–^, mmol/L	20.7 (18.3–22.8)	18.9 (16.4–21.2)	B-HCO_3_^–^ <19.0 mmol/L	1.95 (1.08–3.52)	0.025
B-BE, mmol/L	–5.6 (–9.1 to –3.6)	–8.9 (–12.4 to –6.1)	B-BE < –7.0 mmol/L	2.68 (1.50–4.80)	0.001
B-Lactate, mmol/L	3.0 (1.7–6.5)	5.2 (2.9–9.3)	B-Lactate ≥4.1 mmol/L	1.92 (1.08–3.39)	0.025
SOFA, score	10.0 (9.0–11.0)	11.0 (10.0–12.0)	SOFA score ≥10	3.73 (1.84–7.55)	<0.001
Urine biomarkers (*n* = 195 at admission and *n* = 164 at day 3)					
Admission cystatin C, ng/mL	160 (59–440)	639 (155–1871)	Admission cystatin C ≥291 ng/mL	3.08 (1.71–5.54)	<0.001
Day 3 cystatin C, ng/mL^a^	45 (17–109)	182 (32–929)	Day 3 cystatin C ≥59 ng/mL	3.36 (1.74–6.50)	<0.001
Admission NGAL, ng/mL	106 (37–427)	439 (98–1286)	Admission NGAL ≥219 ng/mL	3.41 (1.88–6.16)	<0.001
Day 3 NGAL, ng/mL^a^	63 (26–145)	287 (109–903)	Day 3 NGAL ≥110 ng/mL	5.65 (2.81–11.30)	<0.001
Admission TIMP-2 × IGFBP7	0.25 (0.04–0.85)	0.65 (0.14–2.26)	Admission TIMP-2 × IGFBP7 ≥ 0.36	2.09 (1.18–3.70)	0.012
Day 3 TIMP-2 × IGFBP7^a^	0.15 (0.06–0.32)	0.24 (0.10–2.49)	Day 3 TIMP-2 × IGFBP7 ≥ 0.18	1.76 (0.93–3.31)	0.079
Outcome					
Hospital RRT	0 (0.0)	8 (9.1)	Treatment with RRT		n.a.
Dead at 6 months	32 (29.9)	56 (63.6)	Death		n.a.
PNO at 6 months	37 (34.6)	59 (67.0)	Poor neurological outcome		n.a.

^a^Data from some patients are missing

Categorical data are presented as number (percent), continuous data with skewed distribution as median (interquartile range), and continuous data with normal distribution or mean (± standard deviation)

Presented *p* values are from univariate Pearson’s Chi square analysis

*AKI* acute kidney injury, *B* whole blood, *BE* base excess, *CA* cardiac arrest, *CI* confidence interval, *CPR* cardiopulmonary resuscitation, *HCO*_*3*_^*–*^ bicarbonate, *IGFBP7* insulin-like growth factor-binding protein 7, *n.a.* not applicable, *NGAL* neutrophil gelatinase-associated lipocalin, *OR* odds ratio, *PNO* poor neurological outcome defined as cerebral performance category 3–5, *ROSC* return of spontaneous circulation, *RRT* renal replacement therapy, *S* serum, *SAPS* simplified acute physiology score, *SOFA* sequential organ failure assessment, *TIMP-2* tissue inhibitor of metalloproteinase 2, *VF/VT* ventricular fibrillation/ventricular tachycardia

## Risk factors for acute kidney injury

Many possible risk factors for AKI were identified in the univariate analysis (Table 1). Urine concentrations of cystatin C, NGAL, and TIMP-2 × IGFBP7 were significantly higher in patients with AKI compared with patients without kidney disease both at admission and day 3, except for TIMP-2 × IGFBP7 at day 3 (Table 1). Parameters excluded from the multivariate analysis were time to ROSC (because of 19 % missing data), as well as bicarbonate and lactate concentrations which were strongly correlated (*r* > 0.7) to base excess levels (in order to avoid co-linearity problems). In multiple logistic regression analysis, urine NGAL levels at day 3 (OR 5.46 (95 % CI 2.65–11.24)), SOFA score at admission day (OR 2.83 (95 % CI 1.24–6.50)) and serum urea concentration at admission day (OR 2.82 (95 % CI 1.12–4.66)) were independent risk factors for AKI. The Hosmer and Lemeshow goodness-of-fit test was not significant, indicating a satisfactory fit of the model (χ^2^ = 10.48, df = 6, *p* = 0.11). In the best predictive model, AuROC was 0.774 (95 % CI 0.700–0.848) indicating a good discriminative ability between patients with and without AKI (Model IV, Table 2).

**Table 2:** Multivariate analysis of risk factors for acute kidney injury, mortality and unfavourable neurological outcome in resuscitated, comatose out-of-hospital cardiac arrest patients

	Covariates	Levels	Adjusted OR (95 % CI)	*p* value*	AuROC (95 % CI) with biomarker	AuROC (95 % CI) without biomarker	*p* value**
Risk factors for acute kidney injury^a^							
Model I	Witnessed CA^c^	No/yes	2.27 (0.85–6.07)	0.104	0.747 (0.667-0.817)	0.719 (0.649-0.790)	0.084
(*n* = 195)	SOFA score day 0	≥/<10	3.08 (1.46–6.48)	0.003			
	Urea day 0	≥/<6.7 mmol/L	2.63 (1.40–4.95)	0.003			
	Admission cystatin C	≥/<291/ng/mL	2.42 (2.29–4.54)	0.006			
Model II	Witnessed CA^c^	No/yes	2.15 (0.80–5.77)	0.128	0.752 (0.682-0.821)	0.719 (0.649-0.790	0.046
(*n* = 195)	SOFA score day 0	≥/<10	2.94 (1.39–6.21)	0.005			
	Urea day 0	≥/<6.7 mmol/L	2.63 (1.40–4.95)	0.003			
	Admission NGAL	≥/<219 ng/mL	2.59 (1.37–4.89)	0.004			
Model III	SOFA score day 0	≥/<10	2.76 (1.25–6.10)	0.012	0.725 (0.644-0.806)		
(*n* = 164)	Urea day 0	≥/<6.7 mmol/L	1.98 (1.00–3.93)	0.049			
	Cystatin C day 3	≥/<59 ng/mL	2.87 (1.45–5.70)	0.003			
Model IV	SOFA score day 0	≥/<10	2.83 (1.24–6.50)	0.014	0.774 (0.700-0.848)		
(*n* = 164)	Urea day 0	≥/<6.7 mmol/L	2.82 (1.12–4.66)	0.024			
	NGAL day 3	≥/<110 ng/mL	5.46 (2.65–11.24)	<0.001			
Risk factors for mortality^a^							
Model V	Initial VT/VF^c^	No/yes	4.70 (2.27–9.74)	<0.001	0.811 (0.751-0.872)	0.790 (0.727-0.852)	0.141
(*n* = 195)	AKI within 3 days	Yes/no	2.83 (1.40–5.69)	0.004			
	SOFA sore day 0	≥/<10	3.40 (1.47–7.88)	0.004			
	Admission cystatin C	≥/<291 ng/mL	2.88 (1.44–5.77)	0.003			
Model VI	Initial VT/VF^c^	No/yes	4.11 (1.99–8.53)	<0.001	0.812 (0.751-0.873)	0.790 (0.727-0.852)	0.131
(*n* = 195)	AKI within 3 days	Yes/no	2.86 (1.43–5.74)	0.003			
	SOFA sore day 0	≥/<10	3.28 (1.43–7.50)	0.005			
	Admission NGAL	≥/<219 ng/mL	2.87 (1.44–5.72)	0.003			
Model VII	Initial VT/VF^c^	No/yes	3.13 (1.45–6.72)	0.004	0.784 (0.713-0.854)		
(*n* = 164)	AKI within 3 days	Yes/no	2.44 (1.16–5.13)	0.019			
	SOFA sore day 0	≥/<10	2.67 (1.14–6.23)	0.024			
	Cystatin C day 3	≥/<59 ng/mL	2.45 (1.17–5.13)	0.018			
Model VIII	Initial VT/VF^c^	No/yes	3.12 (1.45–6.69)	0.004	0.785 (0.713-0.857)		
(*n* = 164)	AKI within 3 days	Yes/no	2.06 (0.95–4.47)	0.069			
	SOFA score day 0	≥/<10	2.88 (1.22–6.84)	0.016			
	NGAL day 3	≥/<110 ng/mL	2.85 (1.32–6.14)	0.008			
Risk factors for unfavourable neurological outcome^a^							
Model IX	Initial VT/VF^c^	No/yes	5.07 (2.40–10.74)	<0.001	0.819 (0.759-0.878)	0.810 (0.750-0.870)	0.264
(*n* = 195)	AKI within 3 days	Yes/no	2.67 (1.32–5.40)	0.006			
	BE day 0	</≥–7 mmol/L	2.07 (1.00–4.26)	0.050			
	SOFA score day 0	≥/<10	2.45 (1.08–5.57)	0.032			
	Admission cystatin C	≥/<291 ng/mL	2.06 (1.00–4.25)	0.050			
Model X^b^	Initial VT/VF^c^	No/yes	4.92 (2.35–10.38)	<0.001		0.810 (0.750-0.871)	
(*n* = 195)	AKI within 3 days	Yes/no	3.03 (1.52–6.02)	0.002			
	BE day 0	</≥–7 mmol/L	2.56 (1.29–5.09)	0.007			
	SOFA score day 0	≥/<10	2.48 (1.11–5.54)	0.027			
Model XI	Initial VT/VF^c^	No/yes	3.42 (1.58–7.39)	0.002	0.778 (0.707-0.849)		
(*n* = 164)	AKI within 3 days	Yes/no	2.45 (1.17–5.12)	0.017			
	BE day 0	</≥–7 mmol/L	2.53 (1.24–5.18)	0.011			
	Cystatin C day 3	≥/<59 ng/mL	2.25 (1.09–4.65)	0.029			
Model XII	Initial VT/VF^c^	No/yes	3.77 (1.69–8.38)	0.001	0.797 (0.729-0.866)		
(*n* = 164)	Fluid balance day 0	≥/<4.45 L/day	2.12 (1.05–4.66)	0.037			
	Urea day 0	≥/<6.7 mmol/L	2.13 (1.03–4.43)	0.043			
	BE day 0	</≥–7 mmol/L	2.21 (1.06–4.62)	0.035			
	NGAL day 3	≥/<110 ng/mL	3.41 (1.65–7.06)	0.001			

^a^TIMP-2 × IGFBP7 had *p* values above 0.05 for predicting acute kidney injury, mortality, and unfavorable neurological outcome at all time points

^b^NGAL at day 0 had a *p* value above 0.05 for predicting poor neurological outcome

^c^Data from some patients are missing

Data are from multivariate logistic regression analysis

*p values for the adjusted odds ratio; **p values from comparing the AuROC with and without biomarkers

*OR* odds ratio, *CI* confidence interval, *AuROC* area under the curve in receiver operating characteristics analysis, *CA* cardiac arrest, *SOFA* sequential organ failure assessment, *NGAL* neutrophil gelatinase-associated lipocalin, *VF/VT* ventricular fibrillation/ventricular tachycardia, *AKI* acute kidney injury, *BE* base excess

Addition of biomarker measurements to clinical parameters significantly increased the discriminating power of AKI in Model II, but not in Model I (Table 2). Cystatin C and NGAL levels at day 3 were significantly better to predict AKI stage 2 or 3 than AKI stage 1 (Additional file 1). The ability to predict AKI was similar for urine NGAL and cystatin C concentrations (Additional file 2).

## Risk factors for mortality and poor neurological outcome

Data from univariate analyses revealed many possible risk factors for mortality and PNO (Table 3 and Table 4). Urine cystatin C and NGAL levels at admission and day 3 were significantly higher in non-survivors compared with survivors (Table 3) and in patients with PNO compared with patients who had good neurological outcome (Table 4). In contrast, urine TIMP-2 × IGFBP7 concentrations were similar for both the considered outcomes at any time point. Time to ROSC, bicarbonate and lactate levels were excluded from multiple regression analyses for the same reasons as in the AKI analysis. Independent risk factors for mortality in multivariate analysis were high NGAL concentrations at admission (OR 2.87 (95 % CI 1.44–5.72)), initial non-shockable rhythm (OR 4.11 (95 % CI 1.99–8.53)), presence of AKI (OR 2.86 (95 % CI 1.43–5.74)) and high SOFA score at admission (OR 3.28 (95 % CI 1.43–7.50)). Independent risk factors for PNO in the multivariate analysis were high cystatin C levels at admission (OR 2.06 (95 % CI 1.00–4.25)), initial non-shockable rhythm (OR 5.07 (95 % CI 2.40–10.74)), presence of AKI (OR 2.67 (95 % CI 1.32–5.40)), low base excess levels at admission (OR 2.07 (95 % CI 1.00–4.26)) and high SOFA score at admission (OR 2.45 (95 % CI 1.08–5.57)). The Hosmer and Lemeshow goodness-of-fit tests were not significant, indicating satisfactory fit of the model for mortality (χ^2^ = 4.04, df = 7, *p* = 0.78) and PNO (χ^2^ = 5.84, df = 8, *p* = 0.67). In the best predictive models, the AuROCs were 0.812 (95 % CI 0.751–0.873) and 0.819 (95 % CI 0.759–0.878) indicating a good discriminative ability between survivors and non-survivors (Model VI, Table 2) in addition to patients with PNO and good neurological outcome (Model IX, Table 2), respectively.

**Table 3:** Univariate analysis of risk factors for mortality in resuscitated, comatose out-of-hospital cardiac arrest patients

	Survivors (*n* = 107)	Non-survivors (*n* = 88)	Risk factor for mortality	Crude OR (95 % CI) for mortality	*p* value
Baseline data					
Age, years	59.1 ± 13.1	61.4 ± 14.1	Age ≥60 years	1.37 (0.77–2.41)	0.283
Weight, kg^a^ (*n* = 166)	83.0 (75.0–93.0)	85.0 (75.0–90.0)	Weight ≥85 kg	1.35 (0.73–2.51)	0.335
Male sex	94 (87.9)	71 (80.7)	Female sex	1.73 (0.79–3.80)	0.167
Witnessed CA^a^ (*n* = 194)	101 (95.3)	68 (77.3)	Unwitnessed CA	5.94 (2.13–16.59)	<0.001
Bystander CPR	94 (87.9)	77 (87.5)	Not bystander CPR	1.03 (0.44–2.44)	0.941
ROSC time, min^a^ (*n* = 158)	19.0 (12.0–29.0)	30.0 (23.0–44.0)	Time to ROSC ≥25 min	3.28 (1.68–6.40)	<0.001
Initial VF/VT^a^ (*n* = 193)	86 (81.1)	42 (48.3)	Not initial VF/VT	4.61 (2.42–8.76)	<0.001
SAPS II, score	68.3 ± 10.4	73.0 ± 10.0	SAPS II score ≥69	1.62 (0.91–2.87)	0.099
Admission day					
Diuresis, L/day	2.03 (1.75–2.84)	1.81(1.43–2.45)	Diuresis <1.93 L/day	1.99 (1.12–3.53)	0.018
Fluid balance, L/day	4.05 (2.37–5.74)	4.75 (3.50–6.39)	Fluid balance ≥4.45 L/day	1.92 (1.08–3.39)	0.025
S-Creatinine, μmol/L	98.0 (84.0–114.0)	107.5 (94.3–140.0)	S-Creatinine ≥101 μmol/L	1.76 (1.00–3.11)	0.051
S-Urea; mmol/L	6.3 (5.1–7.7)	7.3 (5.8–9.7)	S-Urea ≥6.7 mmol/L	1.93 (1.09–3.43)	0.023
B-HCO_3_^–^, mmol/L	20.6 (18.9–22.4)	19.0 (16.6–21.2)	B-HCO_3_^–^ <19.0 mmol/L	2.82 (1.55–5.14)	0.001
B-BE, mmol/L	–5.7 (–8.4 to –3.7)	–8.8 (–12.4 to –6.0)	B-BE < –7.0 mmol/L	3.51 (1.94–6.35)	<0.001
B-Lactate, mmol/L	3.3 (1.7–5.9)	5.2 (2.9–9.3)	B-Lactate ≥4.1 mmol/L	2.48 (1.39–4.43)	0.002
SOFA, score	10.0 (8.0–11.0)	11.0 (10.0–12.0)	SOFA score ≥10	4.26 (2.07–8.75)	<0.001
Urine biomarkers (*n* = 195 at admission and *n* = 164 at day 3)					
Admission cystatin C, ng/mL	160 (57–417)	639 (167–2421)	Admission cystatin C ≥291 ng/mL	3.69 (2.08–6.70)	<0.001
Day 3 cystatin C, ng/mL^a^	42 (18–113)	153 (32–777)	Day 3 cystatin C ≥59 ng/mL	3.77 (1.94–7.34)	<0.001
Admission NGAL, ng/mL	91 (42–334)	506 (152–1322)	Admission NGAL ≥219 ng/mL	4.51 (2.46–8.28)	<0.001
Day 3 NGAL, ng/mL^a^	63 (27–148)	221 (98–843)	Day 3 NGAL ≥110 ng/mL	4.43 (2.25–8.69)	<0.001
Admission TIMP-2 × IGFBP7	0.28 (0.05–1.03)	0.45 (0.11–2.23)	Admission TIMP-2 × IGFBP7 ≥ 0.36	1.76 (1.00–3.11)	0.051
Day 3 TIMP-2 × IGFBP7 ^a^	0.15 (0.07–0.33)	0.21 (0.10–0.61)	Day 3 TIMP-2 × IGFBP7 ≥ 0.18	1.59 (0.84–2.98)	0.151
Outcome					
Hospital RRT	3 (2.8)	5 (5.7)	Treatment with RRT	2.09 (0.48–9.01)	0.314
AKI within 3 days	32 (29.9)	56 (63.6)	Presence of AKI	4.10 (2.52–7.46)	<0.001
PNO at 6 months	8 (7.5)	88 (100.0)	Poor neurological outcome		n.a.

^a^Data from some patients are missing

Categorical data are presented as number (percent), continuous data with skewed distribution as median (interquartile range), and continuous data with normal distribution or mean (± standard deviation)

Presented *p* values are from univariate Pearson’s Chi square analysis

*AKI* acute kidney injury, *B* whole blood, *BE* base excess, *CA* cardiac arrest, *CI* confidence interval, *CPR* cardiopulmonary resuscitation, *HCO*_*3*_^*–*^ bicarbonate, *IGFBP7* insulin-like growth factor-binding protein 7, *n.a.* not applicable, *NGAL* neutrophil gelatinase-associated lipocalin, *OR* odds ratio, *PNO* poor neurological outcome defined as cerebral performance category 3–5, *ROSC* return of spontaneous circulation, *RRT* renal replacement therapy, *S* serum, *SAPS* simplified acute physiology score, *SOFA* sequential organ failure assessment, *TIMP-2* tissue inhibitor of metalloproteinase 2, *VF/VT* ventricular fibrillation/ventricular tachycardia

**Table 4:** Univariate analysis of risk factors for poor neurological outcome in resuscitated, comatose out-of-hospital cardiac arrest patients

	Good neurological outcome (*n* = 99)	PNO (*n* = 96)	Risk factor for PNO	Crude OR (95 % CI) for PNO	*p* value
Baseline data					
Age, years	59.2 ± 16.4	61.0 ± 14.7	Age ≥60 years	1.32 (0.75–2.32)	0.339
Weight, kg^a^ (*n* = 166)	83.0 (75.0–93.3)	85.0 (75.0–90.0)	Weight ≥85 kg	1.28 (0.69–2.36)	0.428
Male sex	87 (87.9)	78 (81.3)	Female sex	1.67 (0.76–3.69)	0.200
Witnessed CA^a^ (*n* = 194)	94 (95.9)	75 (78.1)	Unwitnessed CA	6.58 (2.17–20.00)	<0.001
Bystander CPR	86 (86.9)	85 (88.5)	Not bystander CPR	0.86 (0.36–2.02)	0.722
ROSC time, min^a^ (*n* = 158)	22.5 (12.0–29.0)	30.0 (23.0–40.0)	Time to ROSC ≥25 min	3.16 (1.63–6.10)	0.001
Initial VF/VT^a^ (*n* = 193)	82 (83.7)	46 (48.4)	Not initial VF/VT	5.46 (2.79–10.67)	<0.001
SAPS II, score	67.8 ± 10.4	73.1 ± 9.9	SAPS II score ≥69	2.01 (1.13–3.56)	0.017
Admission day					
Diuresis, L/day	2.03 (1.77–2.86)	1.81 (1.43–2.50)	Diuresis <1.93 L/day	1.98 (1.12–3.50)	0.018
Fluid balance, L/day	3.97 (2.58–5.64)	4.80 (3.46–6.45)	Fluid balance ≥4.45 L/day	2.07 (1.17–3.66)	0.012
S-Creatinine, μmol/L	96.0 (84.0–113.0)	107.5 (94.0–139.3)	S-Creatinine ≥101 μmol/L	1.90 (1.08–3.36)	0.026
S-Urea; mmol/L	6.3 (5.3–7.8)	7.1 (5.7–9.6)	S-Urea ≥6.7 mmol/L	1.90 (1.08–3.36)	0.026
B-HCO_3_^–^, mmol/L	20.6 (18.9–22.6)	19.0 (17.1–21.2)	B-HCO_3_^–^ <19.0 mmol/L	2.58 (1.42–4.71)	0.002
B-BE, mmol/L	–5.6 (–8.2 to –3.6)	–8.5 (–12.0- –6.0)	B-BE < –7.0 mmol/L	4.18 (2.30–7.68)	<0.001
B-Lactate, mmol/L	2.8 (1.6–5.4)	5.1 (3.1–9.1)	B-Lactate ≥4.1 mmol/L	2.68 (1.50–4.77)	0.001
SOFA, score	10.0 (9.0–11.0)	11.0 (10.0–12.0)	SOFA score ≥10	3.66 (1.85–7.24)	<0.001
Urine biomarkers (*n* = 195 at admission and *n* = 164 at day 3)					
Admission cystatin C, ng/mL	166 (55–411)	612 (141–2420)	Admission cystatin C ≥291 ng/mL	3.33 (1.85–6.00)	<0.001
Day 3 cystatin C, ng/mL^a^	45 (17–113)	137 (32–595)	Day 3 cystatin C ≥59 ng/mL	3.22 (1.69–6.13)	<0.001
Admission NGAL, ng/mL	91 (42–315)	497 (144–1286)	Admission NGAL ≥219 ng/mL	4.01 (2.21–7.27)	<0.001
Day 3 NGAL, ng/mL^a^	61 (26–146)	213 (95–755)	Day 3 NGAL ≥110 ng/mL	4.21 (2.18–8.12)	<0.001
Admission TIMP-2 × IGFBP7	0.25 (0.04–1.03)	0.43 (0.12–2.15)	Admission TIMP-2 × IGFBP7 ≥ 0.36	1.74 (0.99–3.08)	0.053
Day 3 TIMP-2 × IGFBP7^a^	0.14 (0.07–0.30)	0.23 (0.10–0.61)	Day 3 TIMP-2 × IGFBP7 ≥ 0.18	1.73 (0.93–3.22)	0.084
Outcome					
Hospital RRT	2 (2.0)	6 (6.3)	Treatment with RRT	3.28 (0.64–16.39)	0.137
AKI within 3 days	29 (29.3)	59 (61.5)	Presence of AKI	3.85 (2.12–6.94)	<0.001
Dead at 6 months	0 (0.0)	88 (91.7)	Death		n.a.

^a^Data from some patients are missing

Categorical data are presented as number (percent), continuous data with skewed distribution as median (interquartile range), and continuous data with normal distribution or mean (± standard deviation)

Presented *p* values are from univariate Pearson’s Chi square analysis

*AKI* acute kidney injury, *B* whole blood, *BE* base excess, *CA* cardiac arrest, *CI* confidence interval, *CPR* cardiopulmonary resuscitation, *HCO*_*3*_^*–*^ bicarbonate, *IGFBP7* insulin-like growth factor-binding protein 7, *n.a.* not applicable, *NGAL* neutrophil gelatinase-associated lipocalin, *OR* odds ratio, *PNO* poor neurological outcome defined as cerebral performance category 3–5, *ROSC* return of spontaneous circulation, *RRT* renal replacement therapy, *S* serum, *SAPS* simplified acute physiology score, *SOFA* sequential organ failure assessment, *TIMP-2* tissue inhibitor of metalloproteinase 2, *VF/VT* ventricular fibrillation/ventricular tachycardia

Addition of biomarker measurements to clinical parameters did not significantly increase the discriminating power of mortality or PNO in Model V, VI, or IX, respectively (Table 2). The ability to predict mortality and PNO was not statistically different when comparing urine NGAL and cystatin C concentrations (Additional files 3 and 4).

## Discussion

Our main finding in this prospective study on resuscitated comatose OHCA patients was that the urine concentrations of cystatin C and NGAL sampled at admission and on day 3 were independent risk factors for AKI, mortality, and PNO. In contrast, TIMP-2 × IGFBP7 levels only predicted AKI in urine samples collected at admission. The discriminating power was not uniformly improved in models combining biomarker concentrations and clinical parameters. Overall outcome was very good, with 51 % of the patients alive with good neurological outcome 6 months post-arrest.

The biomarkers in serum and urine that aim to predict AKI and prognosis have many shortcomings that limit their clinical use [9, 10, 22]. In addition to limited discriminating power they differ in organ specificity and time profile. Cystatin C is produced in all nucleated cells and may be used as a marker of glomerular filtration rate [11]. NGAL is expressed in epithelial cells in different organs, and is considered an inflammatory mediator upregulated in tubular injury [12]. TIMP-2 and IGFBP7 are markers of cell cycle arrest [13]; TIMP-2 probably has kidney-protective properties [13], whereas IGFBP7 may reflect renal haemodynamic alterations [13]. Although their time profiles in urine are not fully clarified, NGAL and cystatin C are elevated approximately 48 h prior to the development of the clinical syndrome of AKI, whereas TIMP-2 and IGFBP7 are thought to predict AKI developing within 12 h [23].

Among the most predictive biomarkers of AKI tested in general ICU patients are cystatin C, NGAL, and TIMP-2 × IGFBP7 measured in blood and/or urine samples [13, 23–26]. In CA patients, there are limited data showing that NGAL measured in blood within 4 h after ROSC is a predictor of AKI [15]. It is therefore not surprising that urine cystatin C, NGAL, and TIMP-2 × IGFBP7 were predictors of AKI in the present study. The finding that TIMP-2 × IGFBP7 was not significantly associated with the development of AKI at day 3 is probably caused by the short half-lives of these markers. Unfortunately, urine cystatin C, NGAL, and TIMP-2 × IGFBP7 levels do not discriminate between CA patients with and without AKI, thereby limiting their clinical utility. However, as shown in Additional file 1, our findings that cystatin C and NGAL at day 3 performed better in predicting moderate to severe AKI (stage 2 or 3) compared with mild AKI (stage 1) is interesting, since worsened AKI severity is associated with an increased need for RRT and reduced survival [27]. Because these biomarkers are good predictors of severe AKI they might be used to forecast the need for RRT after CA, as has been shown in general ICU patients [25].

The biomarkers cystatin C, NGAL, and TIMP-2 × IGFBP7 have been prognostic predictors of both renal recovery and mortality in general ICU patients [25, 28, 29]. Although data from CA patients are sparse, one recent study revealed that NGAL measured in blood within 4 h after ROSC was a predictor of mortality and neurological outcome [15]. In another study, enrolment serum NGAL concentrations in CA patients predicted mortality better than neuron-specific enolase and S100B [14]. In agreement with these findings, we found that urine cystatin C and NGAL, but not urine TIMP-2 × IGFBP7, were statistically associated with mortality and PNO. We might hypothesize that cystatin C and NGAL are influenced by whole-body ischemia and reperfusion injuries, whereas TIMP-2 and IGFBP7 may be more kidney-specific markers.

Several models to predict outcome after CA have been developed [14, 30], and addition of biomarker levels to clinical parameters have been suggested in order to improve the ability to predict [14, 31]. Although we found that urine cystatin C and NGAL levels on admission were risk factors of mortality and PNO, their AKI predictive accuracy was limited, and the prediction was not uniformly improved by adding biomarker concentrations to the clinical parameters. We therefore consider that these predictive models cannot be used in treatment allocation of patients, as no model had a perfect discriminating ability. This is in agreement with the European guidelines for post-resuscitation care that recommends a multimodal strategy with prolonged observation in cases with uncertain outcome [32]. However, the biomarkers might be useful in clinical research involving risk stratification of patients.

The present study has several important limitations. No “gold-standard” definition of AKI exists, and the occurrence of AKI in our study was not strictly according to the KDIGO criteria since we only assessed the first 3 days, and lacked data on body weight in 29 patients. There are also limitations in the measurement of urine biomarkers. Since time of urine sampling was not fully standardized, the variation in the time from CA and ROSC to urine sampling will affect biomarker concentrations. The urine collected as spot samples at admission and could potentially be diluted with urine present in the urinary bladder prior to arrest. This might affect the measured concentrations of biomarkers in our study, and peak values are most likely missed. Moreover, the urine was stored in a refrigerator longer than recommended (i.e., 24 h) and was centrifuged later than recommended (i.e., before freezing). However, as previous studies have revealed a good stability of AKI biomarkers independent of storage time [33, 34] and timing of centrifugation [33, 34], we consider the results to be valid. We also performed a pilot study in 10 ICU patients with and without AKI confirming the stability of urine cystatin C, NGAL, and TIMP-2 × IGFBP7 [21]. Furthermore, we were unable to compare the predictive ability of biomarkers at admission and day 3 since we did not have urine samples from day 3 in 31 patients. We were also unable to include time to ROSC in the multivariate analyses because data were missing in 37 patients; this might be an important co-variate among others not included in our analyses since time to ROSC is a strong predictor of PNO in most studies [32, 35, 36]. Additionally, we have not controlled for the development of AKI when assessing the ability of biomarkers to predict mortality and PNO. Finally, our study had a limited sample size and might also have restricted external validity.

Strengths of the study are that all patients came from the same cohort and time period and were treated according to a standardized treatment protocol documenting good and stable outcome over time [35, 37, 38]. We had clear definitions of risk factors and outcome variables, and tested the biomarkers in a population with a high pre-test probability of the considered outcomes.

## Conclusions

In this observational study of resuscitated comatose OHCA patients, urine cystatin C and NGAL levels at admission and day 3 were independent risk factors for AKI, mortality, and PNO. In contrast, TIMP-2 × IGFBP7 levels only predicted AKI in urine samples collected at admission. Urine cystatin C and NGAL seem to be promising biomarkers that should be explored in future studies, but there are clear limitations in their clinical utility.

## Additional files

Additional file 1: Univariate analysis of risk factors for AKI in subgroups of resuscitated comatose out-of-hospital cardiac arrest patients. Consists of risk factors for AKI in subgroups of patients without AKI (KDIGO stage 0), with mild AKI (KDIGO stage 1) and with severe AKI (KDIGO stage 2–3). (DOCX 24 kb)
Additional file 2: Comparisons of the ability to predict acute kidney injury in out-of-hospital cardiac arrest patients: cystatin C versus NGAL concentrations measured in spot urine. (DOCX 40 kb)
Additional file 3: Comparisons of the ability to predict mortality in out-of-hospital cardiac arrest patients: cystatin C versus NGAL concentrations measured in spot urine. (DOCX 39 kb)
Additional file 4: Comparisons of the ability to predict poor neurological outcome in out-of-hospital cardiac arrest patients: cystatin C versus NGAL concentrations measured in spot urine. (DOCX 27 kb)

## Abbreviations

AKI: Acute kidney injury
AuROC: Area under the receiver operating characteristics curve
CA: Cardiac arrest
CI: Confidence interval
CKD: Chronic kidney disease
CPR: Cardiopulmonary resuscitation
ICU: Intensive care unit
IGFBP7: Insulin-like growth factor-binding protein 7
IQR: Interquartile range
KDIGO: Kidney Disease Improving Global Outcomes
NGAL: Neutrophil gelatinase-associated lipocalin
NORCAST: Norwegian Cardiorespiratory Arrest Study
OHCA: Out-of-hospital cardiac arrest
OR: Odds ratio
PNO: Poor neurological outcome
RCF: Relative centrifugation force
ROC: Receiver operating characteristics curve
ROSC: Return of spontaneous circulation
RRT: Renal replacement therapy
SAPS: Simplified Acute Physiology Score
SD: Standard deviation
SOFA: Sequential Organ Failure Assessment
SOP: Standard operating procedure
TIMP-2: Tissue inhibitor of metalloproteinase-2
TIMP-2 × IGFBP7: Product of the concentrations of TIMP-2 and IGFBP7
TTM: Targeted temperature management
