# Supplementary material for: Urine biomarkers give early prediction of acute kidney injury and outcome after out-of-hospital cardiac arrest
Source: Crit Care. 2016 Oct 5;20:314. doi: 10.1186/s13054-016-1503-2 (PMC5052716; doi:10.1186/s13054-016-1503-2)
Supplement: Additional file 1: — Univariate analysis of risk factors for AKI in subgroups of resuscitated comatose out-of-hospital cardiac arrest patients. Consists of risk factors for AKI in subgroups of patients without AKI (KDIGO stage 0), with mild AKI (KDIGO stage 1) and with severe AKI (KDIGO stage 2–3). (DOCX 24 kb) [file 13054_2016_1503_MOESM1_ESM.docx]

| **Additional file 1: Univariate analysis of risk factors for acute kidney injury in subgroups of resuscitated, comatose out-of-hospital cardiac arrest patients** | | | | | | |
| --- | --- | --- | --- | --- | --- | --- |
|  | **AKI KDIGO**  **stage 0**  **(n=107)** | **AKI KDIGO**  **stage 1**  **(n=52)** | **AKI KDIGO**  **stage 2 or 3**  **(n=36)** | **Risk factor**  **for**  **AKI** | **Crude OR**  **(95 % CI)**  **for AKI^*^** | **p-**  **value^*^** |
| **Baseline data** |  | |  |  |  |  |
| Age, years | 60.0 ± 13.7 | 60.3 ± 13.0 | 60.2 ± 14.2 | Age ≥ 60 years | 1.16 (0.49-2.72) | 0.737 |
| Weight, kg^a^ (n=166) | 80.0 (75.0-90.0) | 84.0 (79.5-100.0) | 85.0 (80.0-94.3) | Weight ≥ 85 kg | 2.20 (0.84-5.76) | 0.105 |
| Male sex | 92 (86.0) | 41 (78.8) | 32 (88.9) | Female sex | 0.47 (0.14-1.60) | 0.218 |
| Witnessed CA^a^ (n=194) | 98 (92.5) | 41 (78.8) | 30 (83.3) | Unwitnessed CA | 0.75 (0.25-2.24) | 0.600 |
| Bystander CPR | 96 (89.7) | 43 (82.7) | 32 (88.9) | Not bystander CPR | 0.60 (0.17-2.11) | 0.421 |
| ROSC time, min^a^ (n=158) | 22.0 (15.0-29.0) | 29.0 (15.0-37.0) | 30.0 (24.0-47.0) | Time to ROSC ≥ 25 min | 2.05 (0.72-6.89) | 0.176 |
| Initial VF/VT^a^ (n=193) | 76 (71.0) | 32 (62.7) | 20 (55.6) | Not initial VF/VT | 1.35 (0.57-3.21) | 0.501 |
| SAPS II, score | 68.2 ± 10.1 | 70.7 ± 9.8 | 76.6 ± 10.3 | SAPS II score ≥ 69 | 2.38 (0.93-6.04) | 0.065 |
| **Admission day** | | | | | | |
| Diuresis, L/day | 2.26 (1.82-3.28) | 1.80 (1.54-2.07) | 1.43 (0.86-1.87) | Diuresis < 1.93 L/day | 2.37 (0.91-6.20) | 0.075 |
| Fluid balance, L/day | 4.01 (2.79-5.77) | 4.46 (3.39-5.24) | 5.26 (3.26-7.19) | Fluid balance ≥ 4.45 L/day | 1.46 (0.61-3.45) | 0.394 |
| S-Creatinine, µmol/L | 94.0 (81.3-105.0) | 108.5 (95.3-138.5) | 140.0 (117.5-194.3) | S-Creatinine ≥ 101µmol/L | 3.88 (1.29-11.61) | 0.012 |
| S-Urea; mmol/L | 6.3 (5.2-7.5) | 7.3 (5.7-8.7) | 9.3 (6.3-12.8) | S-Urea ≥ 6.7 mmol/L | 1.76 (0.71-4.40) | 0.224 |
| B-HCO_3_^-^, mmol/L | 20.7 (18.3-22.8) | 19.4 (17.6-21.5) | 19.0 (16.2-21.0) | B-HCO3^-^ < 19.0 mmol/L | 1.36 (0.58-3.20) | 0.476 |
| B-BE, mmol/L | - 5.6 (-9.1- -3.6) | - 8.0 (-10.0- -6.0) | - 8.9 (-12.7- -6.2) | B-BE < - 7.0 mmol/L | 1.36 (0.56-3.29) | 0.502 |
| B-Lactate, mmol/L | 3.0 (1.7-6.5) | 3.8 (2.7-7.1) | 6.6 (3.7-10.9) | B-Lactate ≥ 4.1 mmol/L | 3.24 (1.28-8.21) | 0.012 |
| SOFA, score | 10.0 (9.0-11.0) | 11.0 (10.0-12.0) | 12.0 (11.0-13.8) | SOFA score ≥ 10 | 1.67 (0.47-5.92) | 0.421 |
| **Urine biomarkers** (n=195 at admission and n=164 at day three) | | | | | | |
| Adm. Cyst C, ng/mL | 160 (59-440) | 463 (108-2042) | 1041 (179-1871) | Adm. Cyst C ≥ 291 ng/mL | 1.76 (0.71-4.40) | 0.224 |
| Day 3 Cyst C, ng/mL^a^ | 45 (17-109) | 93 (26-357) | 461 (81-2781) | Day 3 Cyst C ≥ 59 ng/mL | 3.20 (1.00-10.26) | 0.045 |
| Adm. NGAL, ng/mL | 106 (37-427) | 336 (94-1332) | 600 (176-1094) | Adm. NGAL ≥ 219 ng/mL | 1.50 (0.60-3.77) | 0.390 |
| Day 3 NGAL, ng/mL^a^ | 63 (26-145) | 153 (60-480) | 844 (213-2705) | Day 3 NGAL ≥ 110 ng/mL | 4.67 (1.19-18.35) | 0.020 |
| Adm. [TIMP-2]·[IGFBP7] | 0.25 (0.04-0.85) | 0.47 (0.08-1.30) | 0.76 (0.21-2.84) | Adm. [TIMP-2]·[IGFBP7] ≥ 0.36 | 1.30 (0.54-3.11) | 0.559 |
| Day 3 [TIMP-2]·[IGFBP7]^a^ | 0.15 (0.06-0.32) | 0.21 (0.09-0.60) | 0.34 (0.12-0.86) | Day 3 [TIMP-2]·[IGFBP7] ≥ 0.18 | 1.80 (0.65-5.01) | 0.258 |
| **Outcome** | | | | | | |
| Hospital RRT | 0 (0.0) | 0 (0.0) | 8 (22.2) | Treatment with RRT |  | n.a. |
| Dead at 6 months | 32 (29.9) | 32 (29.9) | 29 (80.6) | Death |  | n.a. |
| PNO at 6 months | 37 (34.6) | 37 (34.6) | 31 (86.1) | Poor neurological outcome |  | n.a. |

Categorical data are presented as number (percent), continuous data with skewed distribution as median (interquartile range) and continuous data with normal distribution or mean (± standard deviation).

Presented p-values are from univariate Pearson’s Chi square analysis. *AKI* acute kidney injury, *KDIGO* kidney disease improving global outcome, *OR* odds ratio, *CI* confidence interval, *n* number,

*CA* cardiac arrest, *CPR* cardiopulmonary resuscitation, *ROSC* return of spontaneous circulation, *VF/VT* ventricular fibrillation/ventricular tachycardia, *SAPS* simplified acute physiology score, *S* serum,

*B* whole blood, *HCO_3_^-^* bicarbonate, *BE* base excess, *SOFA* sequential organ failure assessment, *Adm.* admission, *Cyst* cystatin, *NGAL* neutrophil gelatinase-associated lipocalin, *TIMP-2* tissue inhibitor of metalloproteinase 2, *IGFBP7* insulin-like growth factor-binding protein 7, *RRT* renal replacement therapy, *PNO* poor neurological outcome defined as Cerebral performance category (CPC) 3-5,

*n.a.* Not applicable. ^a^ Data from some patients are missing, ^*^ Values are from comparing AKI KDIGO stage 1 versus AKI KDIGO stage 2 or 3.
