# Supplementary material for: Urine biomarkers give early prediction of acute kidney injury and outcome after out-of-hospital cardiac arrest
Source: Crit Care. 2016 Oct 5;20:314. doi: 10.1186/s13054-016-1503-2 (PMC5052716; doi:10.1186/s13054-016-1503-2)

**Additional file 2:** Comparisons of the ability to predict acute kidney injury in out-of-hospital cardiac arrest patients: Cystatin C versus NGAL concentrations measured in spot urine

Comparison 1: Ability to predict acute kidney injury in urine collected at admission:

Cystatin C (xb1) versus NGAL (xb2)


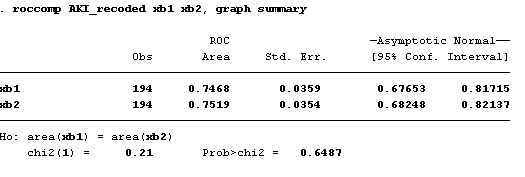


Comparison 2: Ability to predict acute kidney injury in urine collected at day three:

Cystatin C (xb1) versus NGAL (xb2)


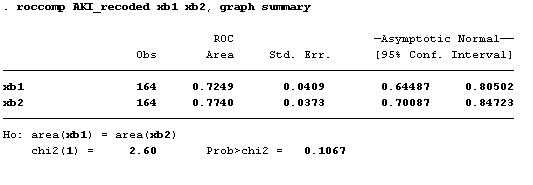

Supplement: Additional file 2: — Comparisons of the ability to predict acute kidney injury in out-of-hospital cardiac arrest patients: cystatin C versus NGAL concentrations measured in spot urine. (DOCX 40 kb) [file 13054_2016_1503_MOESM2_ESM.docx]
